# Extracellular Vesicles as Mediators of Pathophysiology and Disease Progression in Cardiovascular Diseases

**DOI:** 10.3390/ijms27156786

**Published:** 2026-07-29

**Authors:** Melina Tangos, Luca Schneider, Oliver Jarkas, Ibrahim El-Battrawy, Nazha Hamdani

**Affiliations:** 1 Department of Cellular and Translational Physiology, Institute of Physiology, Medical Faculty, Ruhr University Bochum, 44801 Bochum, Germany; melina.tangos@rub.de (M.T.); luca.schneider@rub.de (L.S.); oliverjarkas@gmail.com (O.J.); ibrahim.el-battrawy@ruhr-uni-bochum.de (I.E.-B.); 2 Department of Cardiology and Rhythmology, St. Josef-Hospital, Ruhr University Bochum, 44801 Bochum, Germany; 3 Department of Physiology, Cardiovascular Research Institute Maastricht, University Maastricht, 6200 MD Maastricht, The Netherlands

**Keywords:** cardiovascular diseases, extracellular vesicles, cell communication, biomarker discovery

## Abstract

The rising prevalence of cardiovascular diseases (CVDs) worldwide imposes a substantial economic burden on healthcare systems. Despite major advances in evidence-based therapies and modern healthcare, the demand for novel, time- and cost-effective treatment options continues to grow. Extracellular vesicles (EVs), including apoptotic bodies, microvesicles, and exosomes, are released by numerous cell types and mediate intercellular communication in both physiological and pathological contexts. To date, researchers have amassed compelling evidence for the functional roles of EVs in the development and progression of myocardial disease, supporting their exploration for clinical applications. In this review, we examine the multifunctional roles of EVs from diverse cellular origins in prominent CVD manifestations, highlighting publications of the last eight years and their key findings. They confirm that EVs carry disease-specific molecular cargo, particularly microRNAs, long non-coding RNAs, proteins, and other bioactive molecules, contributing to inflammation, oxidative stress, fibrosis, endothelial dysfunction, and cardiac remodeling, while also serving as promising diagnostic and prognostic biomarkers. Furthermore, emerging preclinical evidence demonstrates the therapeutic potential of engineered or tissue-derived EVs for promoting cardiac repair and limiting adverse cardiac remodeling.

## 1. Introduction

Extracellular vesicles (EVs) were first described about four decades ago in sheep reticulocytes as membrane-bound vesicles carrying transferrin receptors that are externalized during reticulocyte maturation, a process proposed to eliminate reticulocyte-specific membrane proteins [1]. For many years thereafter, EVs were considered membrane debris whose primary role was to dispose of unwanted cellular components, with little biological significance attributed to them [2]. In 1996, however, Raposo et al. demonstrated that EVs can modulate adaptive immunity.

Nowadays, extracellular vesicles are defined as membrane-enclosed vesicles secreted into the extracellular milieu, bounded by a lipid bilayer that encapsulates soluble cytosolic components and nuclear material. EVs can engage membrane receptors via proteins and bioactive lipids inherited from the donor (progenitor) cell, thereby enabling cell-type–specific targeting through source–sink interactions, while also delivering effector molecules directly into recipient cells [3,4]. Reflecting their role as mediators of intercellular communication, EVs are detectable in all body fluids (e.g., blood, saliva, urine, breast milk, cerebrospinal fluid, amniotic fluid, seminal plasma, and bronchoalveolar lavage fluid) [5]. Although essentially all cells can secrete multiple EV populations, the abundance and composition of EV cargo—DNA, RNA, lipids, proteins, and metabolites—are cell-type specific and are shaped by the physiological or pathophysiological state of the source cell, external stimuli, and the molecular pathways governing biogenesis [6].

**Figure 1:**
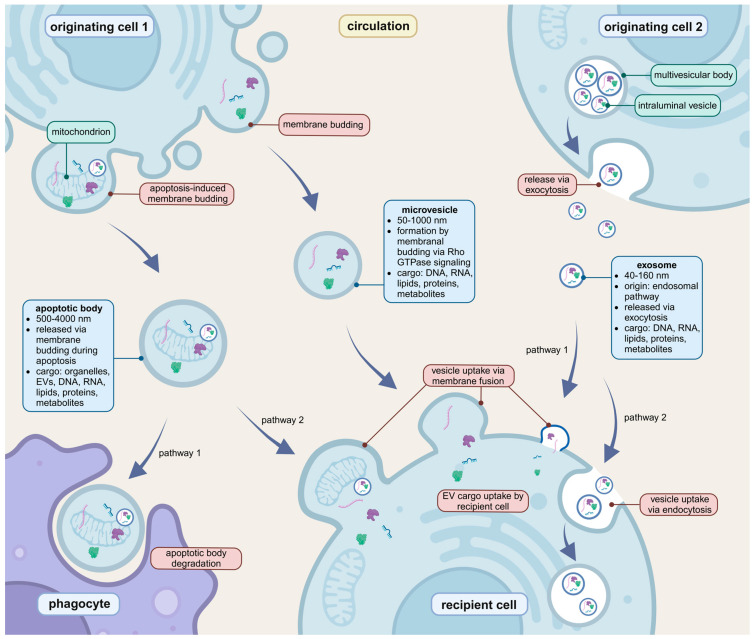
Schematic representation of extracellular vesicle (EV) characteristics, including their biogenesis, release, and uptake by recipient cells via circulation. The smallest EVs, exosomes (**right**), are formed within cells and originate from the endosomal system. Before being released into circulation via exocytosis, they exist in the form of intraluminal vesicles, contained within multivesicular bodies. Larger microvesicles (**middle**) and apoptotic bodies (**left**) are generated by outward membrane budding during physiological and pathophysiological stimuli. All EV types are known to contain bioactive molecules and to contribute to cellular communication by circulating through the bloodstream until their cargo enters new host cells via direct membrane fusion or endocytosis. Abbreviations: EV = extracellular vesicle; DNA = deoxyribonucleic acid; RNA = ribonucleic acid. Created in BioRender. Tangos, M. (2026) https://BioRender.com/3fhfkdq (accessed on 23 July 2026).

Three major EV classes are commonly described: exosomes, microvesicles, and apoptotic bodies (Figure 1) [7]. Exosomes (~40–160 nm) arise along the endosomal pathway as intraluminal vesicles (ILVs) formed by inward budding of endosomal membranes and are released when multivesicular bodies (MVBs) fuse with the plasma membrane [8]. Microvesicles (also termed microparticles or ectosomes; ~50–1000 nm) bud directly outward from the plasma membrane. Their formation involves multiple proteins and is regulated by Rho GTPase–dependent signaling pathways. Like exosomes, microvesicles act as important biological messengers [9,10]. Apoptotic bodies generally are larger than exosomes and microvesicles, with a size range of about 500–4000 nm. Apoptosis is characterized by numerous morphological changes within the cell, including chromatin condensation and fragmentation, and cytoplasmic and nuclear fragmentation. Changes in the cell membrane generate apoptotic bodies that enclose cellular components, including organelles, DNA fragments, RNA, lipids, and proteins [11,12].

According to the “International Society for Extracellular Vesicles”, the general term EV is recommended to describe all vesicle populations that are limited by a lipid bilayer and are not able to replicate themselves [13]. Although exosomes are among the most studied vesicle subtypes in this field, we will use the term EV throughout the manuscript to avoid confusion, given that the publications we cite employ different EV isolation protocols, thereby investigating distinct EV subpopulations.

Cardiovascular diseases (CVDs) represent a global health problem and massive burden for healthcare systems worldwide, with prevalence expected to increase significantly within the next decades [14,15]. Many manifestations of CVD, such as atrial fibrillation (AF), coronary heart disease (CHD), heart failure (HF), and myocardial infarction (MI), exhibit intertwined relationships and the potential to reinforce each other [16,17,18,19,20]. It is widely acknowledged that hypertension, high cholesterol, overweight, obesity, dyslipidemia, lack of physical activity, increasing age, gender, diabetes mellitus (DM), smoking, and alcohol abuse potentiate the risk of developing CVDs [21,22,23]. In this context, EVs have emerged as a promising tool for CVD biomarker research, owing to their potential to trigger and enhance molecular cascades under physiological and pathological conditions, driven by underlying cargo modifications [24,25]. Among the risk factors, DM is particularly relevant to EV-mediated cardiovascular injury [26]. Chronic hyperglycemia and the associated metabolic stress can modify both EV release and cargo composition, thereby altering communication between circulating cells, the vascular endothelium, and the myocardium [27]. In patients with concomitant DM and CVD, these alterations may aggravate endothelial dysfunction [28], thromboembolic activity [29], and myocardial damage [30], while disease-associated EV cargo may reflect the severity of coronary and metabolic abnormalities. Accordingly, DM should be considered a biological modifier of EV function and an important stratification variable when comparing EV-related findings across different CVD manifestations [31]. Beyond DM, the other established risk factors have also been associated with alterations in EV release and molecular cargo. Hypertension has been linked to increased circulating endothelial- and platelet-derived EVs reflecting wall shear stress, inflammation, and vascular dysfunction [32]. Likewise, obesity and dyslipidemia promote the secretion of adipose tissue-derived EVs enriched in cargo that contribute to vascular dysfunction, atherosclerosis, and insulin resistance [33,34]. Furthermore, aging is accompanied by increased release of senescent cell-derived EVs that promote chronic low-grade inflammation and tissue degeneration [35]. Collectively, these findings suggest that EVs respond dynamically to cardiovascular risk factors even before overt disease develops, highlighting their capacity to reprogram recipient cells, orchestrating local and systemic responses to injury and insults.

During the disease state, EVs can induce inflammation, oxidative stress, fibrosis, and apoptosis, significantly contributing to endothelial dysfunction [36,37]. Specifically, endothelial-derived EVs have been identified as a marker indicating endothelial dysfunction, whereas cardiomyocyte-derived EVs show prognostic and diagnostic value for cardiac damage [38,39]. Respectively, cardiomyocyte-derived EVs have been shown to modulate immune response, inflammation, oxidative stress, angiogenesis, cardiac remodeling, fibrosis, autophagy, and metabolism via protein and microRNA (miRNA) delivery, and thus, actively contribute to the development and maintenance of CVDs [38,40].

Interestingly, several studies observed the capability of EV crosstalk between cardiomyocytes and macrophages, able to activate M1 and non-inflammatory M2 macrophage polarization upon specific stimuli (e.g., hypoxia, ferroptosis, or ischemia). The recruitment of M2 macrophages via EV transfer reduced oxidative stress and, consequently, cardiac injury, whereas M1 macrophages led to cardiac cell death, providing further insights into the important modulatory role of EVs and their content [41,42,43].

Although numerous reviews have described the role of EVs in individual CVDs or focused on their diagnostic and therapeutic potential, a comprehensive overview of EV-mediated mechanisms across AF, CHD, MI, and HF remains limited. Previous reviews have rarely addressed how disease subtypes, comorbidities such as diabetes mellitus, differences in EV origin, and methodological variability in EV isolation influence biomarker discovery. In the following paragraphs, we aim to summarize recent important clinical and experimental findings underlining the pathophysiological role of EVs of different origins, alongside benefits arising from EV treatment of patients with different CVD manifestations. For this purpose, the biomedical literature database PubMed was utilized.

## 2. Extracellular Vesicles in Atrial Fibrillation

Atrial fibrillation (AF) is the most common arrhythmia and is characterized by electrical dysfunction, irregular atrial beating, and fibrosis, inflammation, and oxidative stress [44]. Currently, approximately 4.5 million people in Europe and 3 million in the USA are diagnosed with AF, estimated to increase significantly within the next decades [45]. AF predisposes to the development of strokes, HF, MI, thromboembolism, dementia, and even cancer, significantly contributing to higher mortality rates [20]. The role of EVs in the development and perpetuation of AF has been intensively investigated, leading to the identification of typical AF hallmarks associated with EV trafficking.

### 2.1. MicroRNA EV Biomarkers

**Figure 2:**
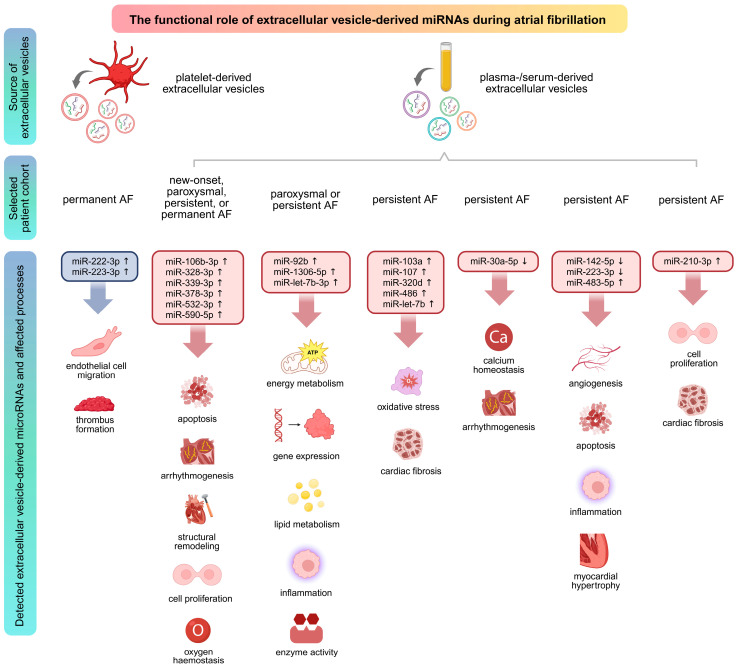
The functional role of extracellular vesicle-derived miRNAs during atrial fibrillation. Abbreviations: AF = atrial fibrillation; miR/miRNA = microRNA; ATP = adenosine triphosphate; O = oxygen; Ca = calcium; ↑ = upregulated; ↓ = downregulated. Created in BioRender. Tangos, M. (2026) https://BioRender.com/xec0yon (accessed on 23 July 2026).

The biological functions of EVs are largely determined by their molecular cargoes—such as miRNAs (Figure 2)—which regulate intercellular communication by modulating signaling pathways in recipient cells [46]. Moreover, it has been elucidated that the amount of EVs released into circulation can be modified under pathological conditions [47]. During AF, the number of EVs in the circulation of affected individuals increases, suggesting enhanced production and release in response to disease-specific stimuli [48,49]. One study found an increase in the number of platelet-derived large EVs (lEVs) in patients with permanent AF. This phenomenon could neither be observed in the non-permanent nor in the non-AF patient group. The lipopolysaccharide-induced activation of isolated platelets triggered the release of lEVs with elevated levels of miRNAs miR-222-3p and miR-223-3p, which impaired endothelial cell migration and potentially contributed to thrombus formation [50]. Siwaponanan et al. isolated EVs from the plasma of AF and sinus rhythm (SR) patients. Six EV-derived miRNAs, namely miR-106b-3p, miR-590-5p, miR-339-3p, miR-378-3p, miR-328-3p, and miR-532-3p, were highly upregulated in AF patients and exert functions in apoptosis, arrhythmogenesis, structural remodeling, cell proliferation, and oxygen hemostasis [48]. Additionally, Wei et al. identified 150 genes that were significantly altered in EVs isolated from the plasma of patients with paroxysmal or persistent AF compared with SR controls. MiRNA target gene enrichment analysis with the top 20 significantly upregulated miRNAs (e.g., miR-92b-3p, miR-1306-5p, and miR-let-7b-3p) in the two AF subgroups revealed their potential influence on gene expression, transport, energy metabolism, lipid metabolism, inflammatory response, and enzyme modification. Notably, a comparison between plasma EVs from patients with paroxysmal versus permanent AF identified 80 differentially expressed genes [51]. The need for disease subclassification was further underscored by the findings of Mun et al., who reported that 45 miRNAs were significantly altered in serum EVs of patients with persistent AF, but not in those with paroxysmal AF, compared with controls with supraventricular tachycardia (SVT), another heart rhythm disorder. In particular, miR-103a, miR-107, miR-320d, miR-486, and miR-let-7b showed log2 fold changes greater than 4.5. The subsequent target gene analysis depicted their role in oxidative stress, fibrosis, signaling pathways, atrial function, and structure [52]. Mun et al. also confirmed the detrimental effects of small EVs originating from the blood of persistent AF patients on cell viability, together with elevated ROS production, unbalanced calcium handling, and upregulation of CaMKII using atrial cardiomyocytes. Quantitative real-time reverse transcription polymerase chain reaction (qRT-PCR) revealed the depletion of miR-30a-5p inside AF EVs. Upon loading miR-30a-5p into the patient EVs, calcium handling normalized, depicting its role in arrhythmogenesis [53]. After high-throughput sequencing, Wang et al. found that plasma-derived EVs from persistent AF patients contain 39 differentially expressed miRNAs compared to those isolated from SR patients. After further qRT-PCR, followed by univariate logistic analysis, the group highlighted 3 miRNAs in the context of AF: miR-483-5p, miR-142-5p, and miR-223-3p. Interestingly, they did not find any significant change in the concentration, and thus, indirectly, the number of EVs between AF and SR. Notably, according to the authors, these findings were inconsistent with several previous reports [54]. Another work identified miR-210-3p as a critical regulator of cell proliferation, reflected by increased numbers in tachypacing atrial cardiomyocytes and serum of AF patients. Knockout of miR-210-3p reduced atrial fibrosis, identifying Angiotensin II (Ang II) and glycerol-3-phosphate dehydrogenase 1-like (GPD1L) as essential mediators of this effect [55].

### 2.2. Long Non-Coding RNA EV Biomarkers

A recent rapid electrical stimulation (RES) experiment with atrial fibroblasts revealed the function of EV-derived lncRNA MALAT1 in suppressing miR-499a-5p expression, thereby upregulating SRY-Box Transcription Factor 6 (SOX6), which is known to regulate cardiac fibrosis. This study proposes that miR-499a-5p promotes fibroblast apoptosis and that MALAT1-containing fibroblast EVs confer protective benefits during AF [56]. Chen et al. explored the role of Ang II-induced AF, providing evidence for the involvement of serum EV-derived long non-coding RNA (lncRNA) MIAT on atrial remodeling. Specifically, the MIAT-miR-485-5p-CXCL10 axis has been implicated in the development of inflammation, oxidative stress, and fibrosis in AF [57].

### 2.3. Protein EV Biomarkers

Ni et al. used serum from patients with paroxysmal AF and healthy donors for label-free mass spectrometry after EV isolation. A total of 39 proteins were significantly overexpressed in AF patient EVs, while 18 proteins showed downregulation. The Gene Ontology (GO) analysis of all differentially expressed proteins revealed their involvement in complement activation, response to oxidative stress, and protein oligomerization. Further protein–protein interaction network analysis highlighted a dense network of protein signaling involved in immune system regulation and protein folding. The group particularly highlighted the downregulation of EV-derived folding proteins eEF1A, PDI, and PIN1, which may contribute to the unfolded protein state often observed in AF, subsequently leading to an increase in oxidative stress [58]. Recent evidence has highlighted the pivotal role of EV protein cargo in driving fibrotic remodeling during AF. Plasma-derived EVs from patients with persistent AF are enriched in FN1 and F13A1, both of which are integral components of inflammatory and profibrotic signaling networks, suggesting EV-mediated activation of the TGF-ß pathway [59]. In addition, EVs may further promote fibrosis through active suppression of the cGMP/PKG signaling pathway, potentially mediated by the PIP/ATP2B2 axis [60]. Moreover, plasma EVs of patients with persistent or paroxysmal AF were enriched in tissue factor, an initiator of the extrinsic blood coagulation cascade, postulating the thrombogenic potential of EVs throughout different stages of AF [49].

### 2.4. Epicardial Adipose Tissue EV Biomarkers

Moreover, adipose tissue-derived EVs have been recognized as critical mediators of interorgan communication, particularly between adipose tissue and the heart. As one of the largest endocrine organs, adipose tissue secretes a diverse array of bioactive molecules, including nucleic acids, proteins, lipids, and adipocytokines, which are transported via EVs to distant organs, such as the myocardium [61]. Recent findings further underline the pivotal role of epicardial adipose tissue (EAT) in the development of AF by exacerbating oxidative stress, myocardial inflammation, fibrosis, and fat infiltration, which in turn promote atrial dysfunction by the transfer of bioactive molecules [62]. Zheng et al. isolated EAT from AF patients and non-AF control patients and performed RNA sequencing to determine the regulatory role of non-coding, circular RNAs (circRNAs) in the context of EAT-promoted AF. Out of 2159 detected circRNAs, 528 were found to be upregulated and 579 downregulated in AF patients. The top upregulated circRNAs were associated with genes involved in inflammation and cell proliferation. Based on prior knowledge of serum EVs and their circRNA content, the group suggested that EVs mediate the transfer of circRNAs from EAT to neighbouring cardiac tissue [63]. This was further confirmed by the study of Ernault et al., in which the authors collected EAT of AF patients to conduct EV isolation and subsequent miRNA analysis. Compared with the control tissue secretome, they found elevated levels of EVs in the EAT of patients, which contained 7 miRNAs that presumably regulate the cardiac resting membrane potential. Especially miR-1-3p and miR-133a-3p were markedly increased in patient EAT-EVs and were therefore selected as overexpressed in neonatal rat ventricular myocytes. Resultantly, the conduction was slowed down via decreased expression of ATP-sensitive inward rectifier potassium channel 2 (Kcnj2) and 12 (Kcnj12), indicating EAT-EV-mediated arrhythmia via miRNA transfer [64]. Shaihov-Teper et al. performed an in-depth analysis of epicardial fat-derived (eFat) EVs from AF and non-AF patients. Not only did the group affirm the presence of ongoing fibrosis, inflammation, and apoptosis within the isolated tissue of AF patients, but also an increase in secreted EVs, harboring myriad cytokines such as interleukin-1α (IL-1α), IL-4, IL-6, and tumor necrosis factor-α (TNF-α), adiponectin, and fibrosis-related miRNAs. On the other hand, they found decreased levels of vascular endothelial growth factor (VEGF), soluble VEGF receptor, and IL-10, indicating a hampered anti-inflammatory response. In contrast to non-AF patients, AF patients exhibited a distinct protein profile in AF eFat-EVs, with proteins associated with fibrosis and hypertrophy. Their wound healing assay revealed faster gap closure when human right atrial mesenchymal stromal cells were incubated with EVs derived from AF patients compared to non-AF. Additionally, prior observations were validated by the fact that only rat hearts injected with AF eFat-EVs developed severe myocardial fibrosis, thereby disrupting normal electrophysiological conduction [65].

### 2.5. Canine In Vivo Models for Atrial Fibrillation

Using a beagle animal model for AF, Yao et al. noticed an increase in exosome release in serum after rapid atrial pacing. Furthermore, several proteins, such as transforming growth factor-β1 (TGF-β1), collagen I/III, and matrix metalloproteinases, were significantly increased inside the atrium after 7 days following pacing, which was guided by an upregulation of exosomal miR-21-5p. The group concluded that miR-21-5p acts as a mediator of fibrosis, especially by modulating its downstream targets metalloproteinase inhibitor 3 (TIMP3) and TGF-β1. The use of exosome inhibitor GW4869 was able to revert the adverse effects, directly linking EV-mediated signaling with the pathophysiology of AF [66]. In another canine model, Liu et al. investigated ferroptosis, a form of cell death triggered by iron, and whether it can be triggered by exosome release during AF. For this purpose, they established an in vivo and in vitro model of rapid atrial pacing, which indeed suggests the involvement of EVs in enhancing fibrosis, electrical, and structural remodeling, ferroptosis, and inflammation, shown to be reversed upon addition of GW4869. EVs isolated from pacing cardiac fibroblasts carry miR-23a-3p, which was identified as a ferroptosis-inducing target [67].

### 2.6. Diabetes Mellitus and Atrial Fibrillation

On another note, the risk for complications during AF was potentiated by concomitant diabetes mellitus (DM), a known risk factor for the development of CVDs and consequently, higher mortality rates [68,69,70]. DM can be seen in approximately 30% of all AF patients, affirming further insights into the molecular mechanisms of this intertwined relationship [71]. To gain a deeper understanding of EV dynamics in this context, Siwaponanan et al. compared EV counts and composition isolated from the plasma of AF patients with and without DM. Indeed, they found an increase in EV numbers in the patients with AF and DM and a reduced expression of miR-126, altogether boosting endothelial dysfunction, thromboembolism, and myocardial damage. Moreover, miR-126 was also found to be significantly downregulated in permanent and persistent AF independent of DM and corresponded with a lower left-ventricle ejection fraction (LVEF) [72].

## 3. Extracellular Vesicles in Coronary Heart Disease

Caused by atherosclerosis, a condition during which arterial plaque formation reduces the blood flow to the myocardium, CHD, also known as coronary artery disease (CAD), has been a prominent CVD manifestation and global health burden for decades [73,74]. Due to the concurrent state of inflammation and ischemia, it favours the development of heart complications, ultimately predisposing to life-threatening conditions, such as strokes and MI [75,76]. To date, numerous publications have collected striking evidence associating EVs with the development and maintenance of CHD, including their role in migration, proliferation, apoptosis, autophagy, angiogenesis, fibrosis, inflammation, and calcification [77].

### 3.1. Inflammatory EV Biomarkers

The state of systemic inflammation and acute inflammatory response was identified as a main trigger of increased EV release into circulation during CHD [78]. Interestingly, upon incubation of human umbilical vein endothelial cells (HUVECs) with serum-derived EVs isolated from CHD patients, the inflammatory markers IL-1β, TNF-α, and ICAM-1 were significantly upregulated, confirming their involvement in inflammatory signaling. Overall, patient EVs reduced cell viability and impaired cell migration and angiogenesis, potentially predisposing to endothelial dysfunction and CHD [79]. Another study not only confirmed the presence of elevated numbers of EVs in the patient serum but also highlighted their potential to induce inflammation and apoptosis in HUVECs. The group identified the circulating EV-derived RNA circ_0001785 as a novel biomarker of atherogenesis that alleviates endothelial injury via miR-513a-5p/TGFBR3 signaling [80].

### 3.2. Circular RNA EV Biomarkers

Using a circRNA microarray dataset based on CHD patient EVs, Tong et al. identified 85 differentially expressed circRNAs, of which circRNA0001785, circRNA0000973, circRNA0001741, and circRNA0003922 were identified as promising biomarkers associated with atherosclerotic plaque formation and CHD diagnosis [81]. Additionally, circRNA0001360 and circRNA0000038, isolated from CHD patient blood EVs, were identified as potential targets, as validated by three machine learning algorithms utilizing the database exoRBase. Analysis of the human blood EV expression profile in exoRBase revealed that 85 circRNAs, 43 lncRNAs, and 312 mRNAs in CHD patient plasma EVs exhibit significant expression changes relative to healthy controls. One group provided evidence for their significance in neutrophil extracellular trap (NET) formation, as well as nucleotide-binding oligomerization domain- (NOD) like receptor signaling pathway. Lipid signaling and atherosclerosis were further identified as important processes modulated during CHD, as indicated by circRNA/lncRNA-miRNA-mRNA regulatory network analysis [82].

### 3.3. MicroRNA EV Biomarkers

A variety of publications substantiate the proof of EV-derived miRNAs and lncRNAs in the pathogenesis of atherosclerosis and CHD. Nigeste et al. proved that EVs isolated from cytokine-stimulated endothelial cells reduced cell proliferation, as evidenced by altered expression of miRNAs let-7, miR-23a, miR-126, miR-302, miR-451, and miR-548 [78]. Specifically, miRNA let-7b-5p was identified as an important EV biomarker in CHD with concomitant hyperglycemia, correlating with the degree of coronary stenosis [83]. In 2020, Zhang et al. reported the overexpression of miR-942-5p, miR-149-5p, and miR-32-5p in patient serum-derived EVs, suggesting their utility in the diagnosis of stable CHD [84]. In line with this research, Han et al. recently reported an increase in miRNA let-7c-5p together with miR-335-3p and miR-652-3p in plasma-derived EVs from patients with stable CHD. Their GO analysis revealed that they are likely involved in CHD-related processes, including arrhythmogenic right ventricular cardiomyopathy, regulation of the actin cytoskeleton, the MAPK signaling pathway, type II diabetes mellitus, and the Hippo signaling pathway [85]. Another publication detected elevated numbers of miR-186-5p in CHD patient EVs, which indicated the degree of stenosis, as well as lipid levels, and the risk for major adverse cardiovascular outcomes [86]. The likelihood of implementing miR-21-5p/3p as an early biomarker for the diagnosis of MI has also been proposed, given that patient serum EV levels positively correlated to serum levels of CRP and LDL-c in CHD patients [87].

### 3.4. Long Non-Coding RNA EV Biomarkers

LncRNA MALAT1 and its target miR-125b were further identified as risk factors for CHD and disease severity in patient plasma. MALAT1 level positively correlated with total cholesterol, low-density lipoprotein cholesterol, TNF-α, IL-1β, IL-6, IL-17, and C-reactive protein, pointing towards its involvement in inflammatory signaling, lipid metabolism, and thus, cardiac dysfunction [88]. MALAT1 has also been detected inside EVs present in serum and therefore represents an intriguing biomarker across different sample types [89]. Further proof of dysregulated lncRNA content of EVs was collected by the group of Hosen et al., who identified an increase in endothelial-specific lncRNA PUNISHER in AF plasma EVs, which plays a crucial role during atherosclerosis by regulating the angiogenic activity of endothelial cells [90].

### 3.5. Protein and Gene EV Biomarkers

In addition to CRP and LDL-c, one group proposed the monitoring of Tenascin-1 for the diagnosis of CHD, as evidenced by its elevated expression in patient serum EVs [91]. Another important molecule during CHD is talin-1 (TLN1). Talin-1 can be found within endothelial cells and has a pivotal role in mediating cell-extracellular matrix (ECM) and cell–cell adhesion, thus protecting the endothelial barrier function [92]. It is known that the disruption of ECM components can exacerbate CVDs, underlining the need for further investigation. The group of Gholipour et al. analysed miRNA datasets from CHD blood EV samples and provided evidence for the miR-182-5p- and miR-9-5p-mediated downregulation of TLN1, which was highly upregulated in patient EVs [93]. Comparing the gene datasets of 6 CHD and 6 donor blood EV samples, one group identified 119 differentially expressed genes, participating in the processes of inflammation, molecular metabolic process, immune system process, and response to stimulus pathways, which can be linked to the development of CVDs. In their dataset, ACSBG1 was the most downregulated and DEFA4 the most upregulated gene [94]. Consistent with this research, another group reported evidence describing CARNS1 and S1PR5 as potential novel plasma EV biomarkers. Their approach revealed that S1PR5 expression was correlated with diabetes, heart rate, apolipoprotein B, fasting blood glucose, triglycerides, total cholesterol, and low-density lipoprotein cholesterol, whilst CARNS1 correlated with uric acid [95].

### 3.6. Urinary and Epicardial Adipose Tissue EV Biomarkers

Whilst blood is certainly the most potent and commonly used source of EVs, some researchers have expanded their work to explore alternative sources. Sharma et al. recently presented the isolation of urinary EVs for biomarker discovery in the context of CHD. Mass spectrometry revealed upregulation of SEMG1/2, IGL, ORM1, AZGP1, HSPG2, SERPINA5, CD59, prosaponin, and gelsolin, whereas TF, UMOD, KNG1, AMBP, and prothrombin were downregulated in urinary patient EVs. The pathway enrichment analysis showed their involvement in neutrophil extracellular trap formation, focal adhesion, complement-coagulation, cholesterol metabolism, renin-angiotensin, and CAD-related signaling pathways [96]. In another approach, the group of Liu et al. analysed the miRNA content of CHD patient-derived epicardial adipose tissue EVs and discovered 5 upregulated (miR-141-3p, miR-183-5p, miR-200a-5p, miR-205-5p, and miR-429) and 2 downregulated miRNAs (miR-382-5p and miR-485-3p), as confirmed via RT-qPCR. GO analysis revealed enrichment in processes related to cell survival, proliferation, differentiation, and apoptosis [97].

## 4. Extracellular Vesicles in Myocardial Infarction

CHD is a well-known contributor to the development of MI, which arises from a coronary artery occlusion caused by atherosclerotic plaque rupture. The resultant blood clot reduces the flow of blood to the affected heart region, triggering the formation of necrotic heart tissue [98]. MI is further associated with adverse cardiac remodeling, ventricular wall thinning, heart chamber dilation, and ultimately loss of cardiac function [99]. These facts underscore the need for early detection, targeting the transition from stable CHD to MI. Concerning EV research, several groups approached the identification of reliable early prognostic markers, as well as utilizing EVs post-MI.

### 4.1. MicroRNA, Gene, and Protein EV Biomarkers

Su et al. identified miR-1915-3p, miR-3656, and miR-4507 as potential biomarkers, as they were downregulated in serum EVs derived from MI patients compared with individuals with CHD [100]. Another novel study implemented a source-tracking analysis to ultimately target heart-derived EVs and their genetic profile inside plasma samples from MI patients. For this purpose, they used single-cell sequencing and identified 752 upregulated and 699 downregulated genes in MI patient EVs compared with the stable CHD group, confirming their modulatory role in the transition from CHD to MI. The 779 genes detected in heart-derived EVs were enriched for processes related to cardiac function, heart development, and cardiac stress response. According to their work, three early-warning biomarkers possessed the potential to predict the probability of MI during CHD: OST4, PKIG, and RPL23, known to be involved in metabolic pathways, antigen processing, and the adipocytokine signaling pathway, respectively [101]. In addition to their diagnostic capabilities, proteomic profiling has revealed that EVs provide biomarkers for MI that are not detectable in plasma alone. This includes proteins such as chymotrypsin C, proto-oncogene tyrosine-protein kinase SRC, and C-C motif chemokine ligand 17, which have been identified as novel markers for MI, further expanding the diagnostic utility of EVs [102]. Another post-mortem study extracted pericardial fluid and plasma from MI patients and a control group and concluded that miR-486-5p might serve as a valuable biomarker for MI prognosis, particularly in patients with high-grade atherosclerotic plaque [103].

### 4.2. The Role of EVs During Post-MI Cardiac Remodeling

Furthermore, EVs have been recognized for their critical role in cardiac remodeling following MI. This remodeling process is marked by an initial inflammatory response followed by scar formation and tissue repair. EVs, which are derived from various cellular origins, play a dual role in this process, balancing pro- and anti-inflammatory as well as pro- and anti-fibrotic effects. This complexity underscores the importance of EVs in orchestrating post-MI repair mechanisms, making them valuable targets for therapeutic intervention [104]. Specifically, large EVs are generated in the infarcted myocardium, mainly by cardiomyocytes and endothelial cells. These EVs are rapidly taken up by infiltrating Ly6C+ monocytes. Unlike small EVs or EVs derived from sham hearts, they increase the release of the pro-inflammatory mediators IL-6, CCL2, and CCL7. This promotes a pro-inflammatory environment in the ischemic myocardium during the early phase after MI [105]. In alignment with these findings, one group demonstrated that plasma EVs isolated from MI patients abrogate TNF-α-induced apoptosis. For this purpose, TNF-α pre-stimulated human cardiac myocytes and human cardiac microvascular endothelial cells were incubated with MI patient EVs. Surprisingly, they observed a reduction in pro-apoptotic caspase-3 activity, which was not observed in cells incubated with healthy donor EVs [106]. The group of Geng et al. noticed a decrease in the level of EV-derived miR-143 within MI patient serum compared to control patients, leading to enhanced angiogenesis and thus cardiac repair via the insulin-like growth factor 1 receptor/nitric oxide signaling pathway [107]. The long intergenic non-coding RNA LIPCAR, identified in EVs isolated from MI patient plasma, has been associated with left ventricular remodeling (LVR) post-MI. Elevated LIPCAR levels in these EVs have been shown to reflect the severity of LVR, positioning LIPCAR as a potential biomarker for early detection and monitoring of cardiac remodeling in MI patients. This discovery highlights the expanding role of EV-contained RNAs in clinical diagnostics [108]. However, research has also revealed that EVs derived from infarcted and failing hearts can carry proneoplastic cargo that accelerates tumor growth. This points to the interplay between cardiac dysfunction and cancer progression mediated by EVs, suggesting the need for careful consideration in their therapeutic use post-MI [109].

Conversely, the therapeutic potential of EVs can be enhanced by modulating their miRNA content. For example, knocking down deleterious miRNAs in progenitor cell-derived EVs has been shown to boost their reparative capabilities. This approach represents a promising therapeutic strategy for improving tissue repair and outcomes after MI [110]. Notably, EVs from cardiac tissue exhibited superior therapeutic effects compared to those from other tissues, suggesting that tissue-specific EVs might be tailored as targeted therapies for MI [111]. Innovative approaches such as the use of EV-functionalized hydrogels have also shown promise in promoting neovascularization post-MI. In one study, an injectable gelatin methacrylate (GelMA) hydrogel was used to retain endothelial-derived EVs at the injury site. This limited EV dispersion and enhanced their local regenerative effects. Histological analysis showed reduced abnormal collagen deposition in the GelMA + EV group. They proposed a synergistic antifibrotic mechanism. Crosslinked GelMA may limit further collagen secretion by resident stromal cells. At the same time, local EV retention supports myocardial and vascular tissue preservation and promotes angiogenesis. Together, these effects may reduce pathological collagen accumulation and adverse ventricular remodeling. This was associated with improved contractility and supports a potential cell-free strategy for cardiac repair [112].

## 5. Extracellular Vesicles in Heart Failure

HF is a heterogeneous, long-term disease with a variety of pathophysiological manifestations and a major cause of elevated hospitalization, morbidity, and mortality rates worldwide. The disease is often accompanied by structural remodeling of the heart, particularly the left ventricle. Several subgroups have been implemented based on the left ventricular ejection fraction (LVEF) and thus, the heart’s capability to pump blood throughout the body [113,114]. The two most investigated types of HF are HF with preserved ejection fraction (HFpEF, LVEF ≥ 50%) and HF with reduced ejection fraction (HFrEF, LVEF ≤ 40%), creating two distinct subpopulations of patients with different clinical profiles [115]. Recent research on EVs has significantly enhanced our understanding of their critical role in HF. EVs are particularly important in HF because they are involved in key pathological processes, including inflammation, fibrosis, and cellular apoptosis. Progress in this field has deepened our insights into how EVs contribute to the progression and potential treatment of HF [116,117].

### 5.1. EV Concentration and Surface Characteristics

Recently, it has been observed that EV counts are reliable prognostic markers of HF grade and survival. According to one group, patients with an EV concentration of less than 5.55 × 10^10^ particles/mL in blood exhibited an increased mortality risk, a phenomenon especially monitored in HF patients requiring intensive care [118]. Suades et al. analysed the surface markers of EVs, focusing on the presence or absence of phosphatidylserine (PS). Phosphatidylserine, a phospholipid typically confined to the inner leaflet of the cell membrane, becomes exposed on the outer surface during cellular stress or apoptosis, serving as a key indicator of cell damage. This study revealed that circulating EVs in chronic HF patients exhibit distinct patterns of PS exposure. Specifically, PS-negative EVs predominantly originate from endothelial cells and connexin-43-rich cells, while PS-positive EVs are more commonly derived from platelets. This differential PS exposure underscores the substantial cellular stress experienced by endothelial and red blood cells in chronic HF, which likely contributes to increased EV shedding and altered cellular communication within the cardiovascular system [119]. The literature also indicates that EV release during HF is induced by myocardial stress and particularly increased in myocytes that undergo stretching in response to filling pressure. Their results further indicate different EV landscapes for HFpEF and HFrEF. HFrEF patient-derived EVs show signs of myocardial injury and myocardial stress, whereas signs of myocardial stress are less abundant in HFpEF patient-derived EVs [120]. Further research into the characteristics of platelet-derived EVs (pEVs) has shown significant differences between chronic HF and acute coronary syndrome (ACS). Specifically, chronic HF patients exhibited higher numbers of EVs that are PS-negative, while ACS patients had a predominance of PS-positive EVs. Moreover, chronic HF patients demonstrated significantly lower numbers of pEVs carrying PECAM and αIIb-integrin epitopes (CD31+/AV+, CD41a+/AV+, and CD31+/CD41a+/AV+), indicating potentially different functional capacities beyond coagulation, such as roles in inflammation and cellular communication [121].

### 5.2. MicroRNA and Circular RNA EV Biomarkers

The role of EV-contained miRNAs and circRNAs in HF has garnered significant attention. These RNAs act as key mediators of pathophysiological processes, including cardiac hypertrophy, fibrosis, and angiogenesis. Their ability to influence intercellular communication makes them promising candidates for both the diagnosis and treatment of HF. The working group of Han et al. analysed the blood of HF patients through next-generation sequencing. The hsa_circ_009743 was identified as a promising marker, over-abundant in HF patient blood and EVs, and its silencing successfully hampered cardiomyocyte apoptosis [122]. Another molecule identified as a key player in the process of apoptosis leading to HF is p53. Its associated miRNAs miR-34a, miR-192, and miR-194 have been evaluated as potential EV biomarkers, remarkably enriched in post-MI patients who developed HF [123]. On the other hand, a reduction in miR-425 and miR-744 within plasma EVs from HF patients has been implicated in the progression of fibrosis and HF [124]. In a clinical context, the potential of adipose-derived stem cells (ADSCs) to modulate plasma EV miRNA dynamics was explored in a study involving patients with ischemic HF. Although ADSC treatment did not significantly alter overall EV concentrations, a notable decrease in miR-126 expression was observed after 12 months in the ADSC-treated group compared to the placebo group. This finding suggests that ADSC therapy may have a long-term impact on specific miRNA profiles, which could be relevant for modulating disease progression in HF. However, the long-term clinical benefits of such miRNA changes need further investigation [125]. Expanding on the diagnostic capabilities of EVs, a liquid biopsy approach was employed to investigate the transcriptomes of plasma-derived EVs in patients with acute decompensated HF (ADHF). This study demonstrated that EV-transcripts could effectively differentiate between HFpEF and HFrEF, highlighting the potential of EV-derived RNA as a biomarker for identifying distinct HF subtypes. The use of EV-transcripts in liquid biopsy represents a promising diagnostic tool, but its translation into routine clinical practice will require validation in larger, diverse patient cohorts [126].

### 5.3. The Therapeutic Potential of EVs for Heart Failure

Zhang et al. investigated the systemic delivery of immortalized cardiosphere-derived EVs (imCDCevs) in a rat model of HF with preserved ejection fraction (HFpEF) and associated atrial fibrillation (AF). The study demonstrated that HFpEF-verified animals had significantly higher AF inducibility than controls. However, treatment with imCDCevs effectively reversed adverse electrical remodeling, including the restoration of action potential duration to control levels and the reorganization of connexin. Moreover, the EV treatment reduced AF inducibility and attenuated major pathological drivers such as fibrosis, inflammation, and oxidative stress. Importantly, these beneficial effects occurred without altering blood pressure or diastolic function, suggesting that imCDCevs might offer a novel therapeutic approach for managing AF in HFpEF patients [127]. In another promising development, recent research on EVs derived from human adult cardiomyocytes (CMs) has shown significant potential in treating cardiac fibrosis, a common feature of HF characterized by excessive ECM deposition and impaired cardiac function. EVs from CMs were found to decrease fibroblast activation markers and ECM accumulation in TGF-β-activated human cardiac fibroblasts, leading to reduced fibrosis and improved cardiac function in an animal model. This antifibrotic effect was closely linked to the specific miRNA cargo of the EVs, highlighting their potential as a targeted therapy for cardiac fibrosis [128]. A recent study also underscored the pathological impact of EVs in HF and fibrosis under conditions of pressure overload. In a mouse model of HF induced by transverse aortic constriction (TAC), the administration of tipifarnib, an EV biogenesis inhibitor, significantly reduced circulating EVs and improved cardiac function. Tipifarnib also altered the miRNA profile of circulating EVs, particularly by upregulating miR-331-5p, which inhibits fibroblast-to-myofibroblast transition, a critical process in fibrosis development [129].

## 6. Recent Advances in the Therapeutic Usage of EVs

Recent years have witnessed remarkable advances in EV research, driven by improvements in isolation techniques, high-throughput omics technologies, and a growing understanding of EV biology. These findings have accelerated the development of therapeutic, stem-cell-derived, and engineered EVs that benefit from proper in vivo stability, optimized cargo loading, and enhanced targeting capability, thus representing an intriguing new vessel for a cell-free drug delivery system [130]. Their potential use in clinical routine has been recently emphasized by several preliminary animal model studies. One of these approaches utilizes the stem cell-derived exosome nebulization therapy (SCENT). In their experimental setup, MI mice inhaled lung spheroid cell-derived exosomes for 10 min over a time frame of 7 days, which led to their rapid accumulation in the lungs. Interestingly, after 60 min, a notable rise in the abundance of those exosomes could be observed within the heart, which was enhanced by repeated exposure. In their 42-day study timeframe, they observed a higher survival rate of MI mice in the SCENT group compared to the control groups, highlighting the exosomal miRNA cargo as important modulators [131]. An additional novel, proposed strategy includes an electroactive patch for wirelessly and controllably generating EVs (ePOWER). This patch harboured M2-type electrosensitive macrophages that served as EV donors after its cardiac implantation in MI rats. According to their results, the ePOWER stimulation led to a 2400% increase in EV production compared to unstimulated cell culture controls and effectively reduced fibrosis in the animal hearts. Moreover, their histological examination revealed no evidence of tissue toxicity following electrical stimulation, supporting its potential as a promising avenue for future research [132]. In another study conducted over 3 months, the intracoronary delivery of cardiac progenitor cell EVs via instillation effectively reduced infarct size and improved left-ventricular function in pigs. However, whether these results can be efficiently translated into human therapy, especially in the face of comorbidities, remains elusive [133]. Overall, due to remaining safety concerns and lack of robust experimental long-term validation, the transition from animal to human studies has yet to be established [134]. A novel, promising frontier that may close the gap is the use of artificial intelligence and machine learning (ML) to overcome current limitations, particularly in data analysis and interpretation. The key aspect is that ML allows a rapid analysis of complex EV multi-omics datasets, thereby furthering biomarker discovery, disease outcome predictions, and precision medicine [135,136].

## 7. Conclusions and Limitations

EVs have emerged as important mediators of cell-to-cell communication and are involved in the pathophysiology of various CVDs, including AF, CHD, HF, and MI. The exploration of EVs in this context has revealed their vast and multifaceted potential. They contribute to cardiac remodeling, arrhythmia, angiogenesis, atherosclerosis, cell migration, apoptosis, ferroptosis, oxidative stress, inflammation, and fibrosis, exacerbating endothelial and cardiac dysfunction, and ultimately promoting the progression of CVDs. Among the numerous EV-associated biomarkers that have been identified so far, several stand out because of their reproducibility, correlation with disease severity, and potential diagnostic or prognostic value. As presented in Table 1, EV-derived miRNAs, particularly miR-21 (fibrosis, inflammatory signaling), miR-126 (endothelial and myocardial protection), miR-223 (platelet activation, thrombosis), miR-486 (atherosclerotic plaque development, cardiac remodeling), and miR-let-7 (coronary stenosis, metabolism, cardiac remodeling), may represent promising candidates as they were detected across different CVD manifestations or by multiple independently conducted studies. In addition, the lncRNA MALAT1 has shown strong associations with fibrosis in AF and CHD severity, based on its modulatory effect on miRNAs. Regarding protein biomarkers, tissue factor, Tenascin-1, and EV surface characteristics, including phosphatidylserine exposure and total EV concentration, have demonstrated prognostic potential. Despite these encouraging findings, their routine clinical application remains elusive.

**Figure 3:**
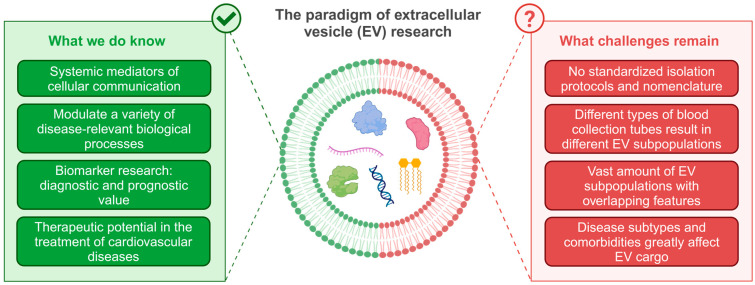
The paradigm of extracellular vesicle (EV) research. The current academic discourse and knowledge compared to remaining obstacles and challenges for future prospects. Created in BioRender. Tangos, M. (2026) https://BioRender.com/yj7op3h (accessed on 23 July 2026).

On the other hand, the exploitation of EVs as therapeutic drugs for CVDs is advancing, even in the face of several drawbacks yet to be overcome (Figure 3). Considering that tissues, organs, blood cells, and even bacteria contribute to the production and release of EVs, it is prudent to underline their complexity in the circulatory system [137]. It is still impossible to segregate diseased EVs from healthy EVs within a patient, and the lack of standardized EV isolation protocols generates a heterogeneous EV research landscape [138]. This is best reflected by contradictory reports of miRNA expression trends, such as the herein presented miR-223-3p. This likely reflects variations in patient cohorts, AF subtype, EV isolation method, source material, and analysis platform. Therefore, as discussed in this review, it is essential to implement a more stringent patient selection process based on disease subtypes and comorbidities, considering that EV biomarkers may only be present under highly specific conditions. Developing fixed guidelines for EV isolation should be a key goal in the near future, particularly given the delicate nature of miRNA and lncRNA biomarker research [139]. Furthermore, the type of blood collection tube greatly impacts EV composition and number. Nanoparticle tracking analysis and mass spectrometry confirmed a higher abundance of overall EV number, particularly platelet-derived EVs, in serum compared with plasma, significantly altering the results [140]. And yet, despite all existing challenges, EVs represent a promising frontier in the management and treatment of CVDs and their complications.

## Figures and Tables

**Table 1:** List of recently explored extracellular vesicle biomarkers in the course of cardiovascular diseases and involved biological processes/pathways. Abbreviations: AF = atrial fibrillation, CHD = coronary heart disease, MI = myocardial infarction, HF = heart failure, EV = extracellular vesicle, EAT = epicardial adipose tissue, ↑ = upregulated, ↓ = downregulated.

Disease	Source	Biomarker	Function/Affected Pathways	Literature
**AF**	platelet EVs	miR-222-3p ↑ miR-223-3p ↑	endothelial cell migration ↓	Zietzer et al., 2022, [50]
	plasma EVs	miR-328-3p ↑ miR-339-3p ↑ miR-378-3p ↑ miR-532-3p ↑ miR-590-5p ↑ miR-106b-3p ↑	apoptosis, arrhythmogenesis, structural remodeling, cell proliferation, oxygen hemostasis	Siwaponanan et al., 2022, [48]
	plasma EVs	miR-let-7b-3p ↑ miR-92b-3p ↑ miR-1306-5p ↑	gene expression, transport, energy metabolism, lipid metabolism, inflammatory response, enzyme modification	Wei et al., 2020, [51]
	serum EVs	miR-let-7b ↑ miR-103a ↑ miR-107 ↑ miR-320d ↑ miR-486 ↑	oxidative stress, fibrosis, signaling pathways, atrial function	Mun et al., 2019, [52]
	blood EVs	miR-30a-5p ↓	arrhythmogenesis ↑	Mun et al., 2022, [53]
	plasma EVs	miR-142-5p ↓ miR-223-3p ↓ miR-483-5p ↑	angiogenesis, inflammation, myocardial hypertrophy, apoptosis	Wang et al., 2019, [54]
	plasma EVs	miR-126 ↓	angiogenesis ↓ left-ventricle ejection fraction ↓	Siwaponanan et al., 2023, [72]
	serum EVs	39 proteins ↑ 18 proteins ↓	complement activation, response to oxidative stress, protein oligomerization, immune system regulation, protein folding	Ni et al., 2021, [58]
	plasma EVs	FN1 ↑ F13A1 ↑	inflammation, fibrosis	Zhang et al., 2026, [59]
	EAT EVs	miR-1-3p ↑ miR-133a-3p ↑	arrhythmia ↑	Ernault et al., 2023, [64]
	EAT EVs	IL-1α ↑ IL-4 ↑ IL-6 ↑ TNF-α ↑ adiponectin ↑ VEGF ↓ soluble VEGF receptor ↓ IL-10 ↓	inflammation ↑ anti-inflammatory response ↓	Shaihov-Teper et al., 2021, [65]
	serum EVs	miR-210-3p ↑	atrial fibrosis ↑	Hao et al., 2022, [55]
	cardiac fibroblast EVs	MALAT1↑ SOX6↑ miR-499a-5p↓	apoptosis↓	Chuang et al., 2024, [56]
	serum EVs	lncRNA MIAT ↑	atrial fibrosis ↑ inflammation ↑ oxidative stress ↑	Chen et al., 2021, [57]
	serum EVs	TGF-b1 ↑ collagen I/III ↑ MMPs ↑ miR-21-5p ↑	fibrosis ↑	Yao et al., 2021, [66]
	cardiac fibroblast EVs	miR-23a-3p ↑	ferroptosis ↑	Liu et al., 2022, [67]
**CHD**	serum EVs	IL-1β ↑ TNF-α ↑ ICAM-1 ↑	inflammation ↑ cell viability ↓ cell migration ↓ angiogenesis ↓	Zhang et al., 2021, [79]
	plasma EVs	circ_0001785 ↓	atherogenesis ↓	Tong et al., 2023, [80]
	plasma EVs	circRNA0000973 ↓ circRNA0001741 ↓ circRNA0001785 ↓ circRNA0003922 ↓	plaque formation and stability	Tong et al., 2023, [81]
	plasma EVs	circRNA0000038 ↓ circRNA0001360 ↑	regulation of cell function, inflammation	Zhang et al., 2024, [82]
	endothelial cell EVs	miR-let-7 ↑ miR-23a ↓ miR-126 ↓ miR-302 ↑ miR-451 ↓ miR-548 ↓	cell proliferation ↓ cell permeability angiogenesis ↓	Carter et al., 2022, [78]
	serum EVs	miR-let-7b-5p ↑	coronary stenosis ↑	Han et al., 2023, [83]
	serum EVs	miR-32-5p ↑ miR-149-5p ↑ miR-942-5p ↑	signal transduction, positive regulation of cell migration, angiogenesis	Zhang et al., 2020, [84]
	plasma EVs	miR-let-7c-5p ↑ miR-335-3p ↑ miR-652-3p ↑	arrhythmogenic right ventricular cardiomyopathy, regulation of actin cytoskeleton, MAPK signaling pathway, type II diabetes mellitus, Hippo signaling pathway	Han et al., 2023, [85]
	serum EVs	miR-186-5p ↑	atherosclerosis ↑	Ren et al., 2024, [86]
	serum EVs	miR-21-5p/3p ↑	inflammation, lipid metabolism	Sahebi et al., 2024, [87]
	serum EVs, plasma	lncRNA MALAT1 ↑	inflammation, lipid metabolism, NET formation, cardiac dysfunction	Lv et al., 2021, [88] Tello-Flores et al., 2020, [89]
	plasma EVs	lncRNA PUNISHER ↑	angiogenesis ↑	Hosen et al., 2021, [90]
	serum EVs	Tenascin-1 ↑	atherosclerosis, endothelial differentiation	Gholipour et al., 2022, [91]
	serum EVs	miR-9-5p ↑ miR-182-5p ↑ Talin-1 ↓	cardiac differentiation, atherosclerosis, inflammation, cardiac remodeling	Gholipour et al., 2022, [93]
	plasma and serum EVs	DEFA4 ↑ ACSBG1 ↓	lipid metabolism, immune response, response to stress	Gholipour et al., 2022, [94]
	plasma EVs	CARNS1 ↓ S1PR5 ↑	atherosclerosis, inflammation	Xiong et al., 2022, [95]
	urine EVs	SEMG1/2 ↑ ORM1 ↑ HSPG2 ↑ SERPINA5 ↑ TF ↓ KNG1 ↓ UMOD ↓ prothrombin ↓	neutrophil extracellular trap formation, focal adhesion, complement-coagulation, cholesterol metabolism, renin-angiotensin, CAD-related signaling pathways	Sharma et al., 2024, [96]
	EAT EVs	miR-141-3p ↑ miR-183-5p ↑ miR-200a-5p ↑ miR-205-5p ↑ miR-429 ↑ miR-382-5p ↓ miR-485-3p ↓	cell survival, proliferation, differentiation, apoptosis	Liu et al., 2022, [97]
**MI**	serum EVs	miR-1915-3p ↓ miR-3656 ↓ miR-4507 ↓	cardiac remodeling, platelet aggregation, heart function	Su et al., 2020, [100]
	heart EVs	OST4 ↑ PKIG ↓ RPL23 ↑	metabolic pathways, antigen processing, adipocytokine signaling pathway	Jin et al., 2024, [101]
	plasma EVs	miR-486-5p ↑	atherosclerotic plaque formation	Kim et al., 2024, [103]
	plasma EVs	CCL17 ↓ CTRC ↓ SRC ↓	atherosclerosis, inflammation, leukocyte migration, platelet activation	Gidlöf et al., 2019, [102]
	serum EVs	miR-143 ↓	angiogenesis ↑ cardiac repair ↑	Geng et al., 2020, [107]
	plasma EVs	LIPCAR ↑	cardiac remodeling ↑	Turkieh et al., 2024, [108]
**HF**	serum EVs	EV number < 5.55 × 10^10^ particles/mL	mortality risk ↑	Schöler et al., 2023, [118]
	plasma EVs	PS^−^ endothelium EVs ↑ PS^−^ connexin-43-rich EVs ↑	endothelial stress ↑ myocardial stress ↑	Suades et al., 2023, [119]
	platelet EVs	PS^−^ EVs ↑ PECAM epitope ↓ αIIb-integrin epitope ↓	Inflammation, cellular communication	Vilella-Figuerola et al., 2023, [121]
	plasma EVs	hsa_circ_0097435 ↑	cardiomyocyte apoptosis ↑	Han et al., 2020, [122]
	serum EVs	miR-34a ↑ miR-192 ↑ miR-194 ↑	potential prediction factors for cardiac fibrosis, LV dysfunction	Zhou et al., 2020, [123]
	plasma EVs	miR-425 ↓ miR-744 ↓	fibrosis ↑	Wang et al., 2018, [124]

## Data Availability

No new data were created or analyzed in this study. Data sharing is not applicable to this article.
